# High-pressure synthesis and crystal structure analysis of PbTeO_4_, a UV transparent material†

**DOI:** 10.1039/d4dt02697g

**Published:** 2024-10-31

**Authors:** Michael Hladik, Armin Penz, Felix R. S. Purtscher, Thomas S. Hofer, Gunter Heymann, Matthias Weil

**Affiliations:** a Department of General, Inorganic and Theoretical Chemistry, Universität Innsbruck, Center for Chemistry and Biomedicine Innrain 80-82 6020 Innsbruck Austria Gunter.Heymann@uibk.ac.at; b Institute for Chemical Technologies and Analytics, Division of Structural Chemistry, TU Wien Getreidemarkt 9/E164-05-1 1060 Vienna Austria Matthias.Weil@tuwien.ac.at

## Abstract

Using the additional parameter pressure (Walker-type multianvil device), the lead(ii) oxidotellurate(vi) PbTeO_4_ was synthesized at conditions of 8 GPa and 750 °C, and for the first time its crystal structure was determined using single-crystal X-ray diffraction data. PbTeO_4_ crystallizes with four formula units in the monoclinic space group *I*2/*a* with unit cell parameters *a* = 5.4142(4), *b* = 4.9471(4), *c* = 12.0437(11) Å, *β* = 99.603(3)°, and *V* = 318.07(5) Å^3^. UV-Vis measurements revealed UV transparency down to 200 nm. From the diffuse reflectance data experimental band gaps (*E*_g(direct)_ = 2.9 eV/*E*_g(indirect)_ = 2.8 eV) were determined and compared with calculated values. Temperature-dependent X-ray powder diffraction and complementary thermal analysis measurements revealed a stability range of PbTeO_4_ up to 625 °C. Additionally, theoretical calculations at DFT level of theory were carried out to obtain the electronic band structure, X-ray powder diffraction patterns, IR/Raman vibrational spectra and Mulliken partial charges. The electron localization function (ELF) was visualized to emphasize the presence of the electron lone pair *E* in the coordination sphere of the Pb^II^ atom.

## Abstract 2

Crystal structure of PbTeO_4_ with presumed position of the Pb^II^ lone-pair electrons (turquoise spheres). The electron localization function was used to emphasize the presence of the electron lone pair *E*.

## Graphical abstract

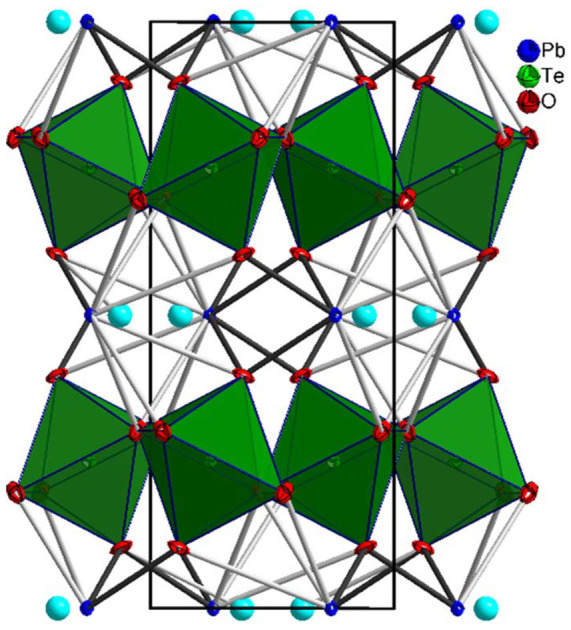

## Introduction

PbTe is a narrow gap semiconductor and an important thermoelectric material for mid-range temperature (600–800 K) applications.^1,2^ Its function is diminished by surfacial oxidation products that can form under atmospheric working conditions.^3^ Hence, it is of importance to have knowledge on the formation conditions, stability ranges and crystal structures of phases in the system Pb–Te–O,^4–7^ for which the following ternary compounds have been structurally determined so far: three polymorphs of Pb^II^Te^IV^O_3_ (α-,^8,9^ β-,^10,11^ γ-^12^), Pb^II^Te^IV^_5_O_11_,^13^ Pb^II^_2_Te^IV^_3_O_8_,^14,15^ Pb^II^_2_Te^VI^O_5_ ^16^ and Pb^II^_5_Te^VI^O_8_.^16^ PbTeO_4_ has been reported previously.^17–19^ It is considered as the thermodynamically most stable compound that forms by oxidizing PbTe under a low-pressure oxygen atmosphere at 623 K,^20^ and was identified as one of the possible oxidation products by cathodoluminescence, TOF-SIMS and Rutherford backscattering spectroscopy.^21^ Nevertheless, structural details of PbTeO_4_ are limited to unit cell parameters.^22^

Apart from the necessity to identify possible oxidation products of PbTe, compounds of divalent lead are in general interesting from a crystal-engineering point of view. The presence of the 6s^2^ free electron pair *E* located at the Pb^II^ atom is in the majority of cases stereochemically active^23^ and consequently promotes an asymmetrical coordination sphere. The resulting off-centred coordination polyhedra exhibit either holo- or hemidirected ligands^24^ and are considered as the driving force for the possible formation of crystal structures without an inversion center. The latter is a pre-condition for physical phenomena like piezoelectricity or non-linear optical properties. Moreover, materials with non-centrosymmetric and polar crystal structures are qualified to show pyroelectricity and/or ferroelectricity.^25^ Thus, new Pb^II^ compounds are possible candidates to comply with the required crystallographic criteria for such materials.

The two points mentioned above motivated us to study formation conditions and the crystal structure of PbTeO_4_, under special attention whether the synthesized material corresponds to Pb^II^Te^VI^O_4_ or to Pb^IV^Te^IV^O_4_, a point that remained open since the first but preliminary structural study of this material.^22^

## Experimental

### High-pressure/high-temperature synthesis

PbTeO_4_ was synthesized by high-pressure/high-temperature multianvil synthesis starting from a stoichiometric mixture of PbO_2_ (purity >95%, Honeywell Fluka) and TeO_2_ (purity >98%, TCI Deutschland GmbH). A platinum capsule containing the finely ground reaction mixture (approx. 65 mg per experiment) was placed in the 18/11-assembly crucible made of hexagonal boron nitride (HeBoSint® P100, Henze BNP GmbH, Kempten, Germany). The crucible was embedded in a MgO octahedron (18 mm MgO octahedra doped with 5% Cr_2_O_3_, Ceramic Substrates & Components Ltd, Newport Isle of Wight, United Kingdom) surrounded by eight tungsten carbide cubes (HM-type ha-7% Co, Hawedia, Marklkofen, Germany) and was placed inside the Walker-type module. After compression to the final pressure of 8.0 GPa (250 bar oil-pressure) with a ramp of 72 bar oil-pressure per hour, the sample was heated to 750 °C within 10 minutes. Subsequently, the corresponding temperature was kept constant for 15 min and then continuously lowered to 600 °C for 45 min in order to obtain a better crystal quality. As soon as the heating process was terminated by a quenching step, the decompression of the press took place with a ramp of 24 bar oil-pressure per hour. The recovered MgO octahedron was broken apart, and the sample was carefully separated from the surrounding crucible and capsule materials. PbTeO_4_ was gained as colorless to light yellow air-stable crystals. Further information about the multianvil technique and constructions of the various assemblies can be found in numerous references.^26–28^

### X-ray powder diffraction

**Fig. 1:**
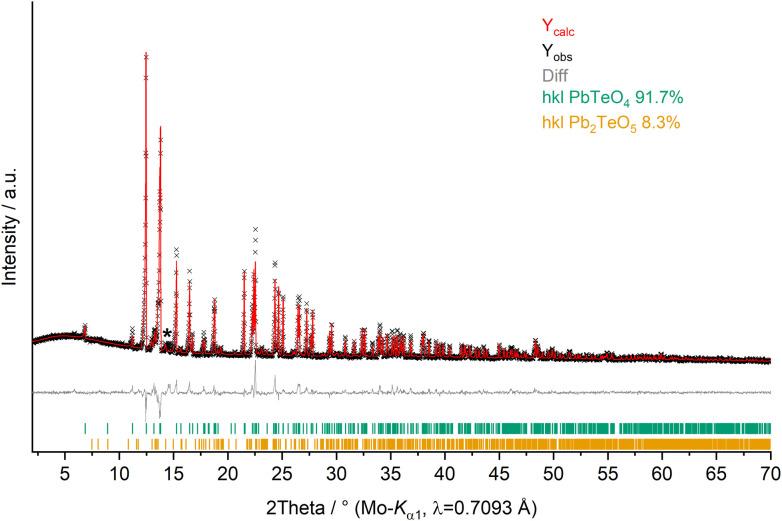
XRPD pattern (Mo-K_α1_ radiation) and Rietveld refinement of PbTeO_4_ (*R*_exp_ = 1.91%, *R*_wp_ = 3.68%, *R*_p_ = 2.46% and GoF = 1.92). Pb_2_TeO_5_ ^16^ was refined as side phase, and an asterisk marks an unassignable reflection.

X-ray powder diffractometry (XRPD) of PbTeO_4_ was carried out both at ambient temperature and temperature-dependent up to maximum temperatures of 950 °C on a STOE Stadi P powder diffractometer (STOE & Cie GmbH, Darmstadt, Germany) in transmission geometry using the WinXPOW software package.^29^ Ge(111) monochromatized Mo-Kα_1_ X-rays (*λ* = 0.7093 Å) were applied to the sample mounted between two thin acetate foils with vacuum grease; diffraction intensities were collected from a Mythen-2 1K microstrip detector with 1280 strips (Dectris AG, Baden-Daettwil, Switzerland). The data collection was performed in the 2Theta range of 2.0–70° with a step size of 0.015° and an exposure time of 25 s per step. Fig. 1 shows the Rietveld refinement plot performed with Diffracplus-Topas (Bruker AXS, Karlsruhe, Germany). As a starting model, the structure data from the single-crystal structure analysis reported here was used, and the peak shapes were modelled using modified Thompson-Cox-Hastings pseudo-Voigt profiles.^30,31^ Instrumental contributions on reflection profiles were corrected from the refinement of a LaB_6_ standard.^32^ The background was fitted with Chebychev polynomials up to the 8^th^ order. Unit cell parameters comparable to those determined by single-crystal X-ray diffraction were obtained (see Table 1). For temperature-dependent measurements, the sample was filled in open 0.3 mm Mark capillaries and placed in a STOE high-temperature furnace. The furnace was heated with 50 °C min^−1^ from room temperature to 950 °C with steps of 50 °C (100–300 °C) and 25 °C (300–950 °C). After each temperature step, a pattern was recorded in the region 9–32° 2Theta.

**Table 1:** Data collection and refinement details

Formula	PbTeO_4_	*μ*/mm^−1^	61.861
*M*_r_	398.79	X-ray density/g cm^−3^	8.328
Temp./°C	25	*θ*_min_ − *θ*_max_/°	3.431–42.471
Crystal dimension/mm^3^	0.03 × 0.02 × 0.005	*h*	−10 to 10
Crystal color	Light-yellow	*k*	−9 to 9
Crystal form	Plate	*l*	−22 to 22
Space group, No.	*I*2/*a*, 15	Measured reflections	10 641
Formula units *Z*	4	Independent reflections	1243
Powder data			
*a*/Å	5.41173(10)		
*b*/Å	4.94608(9)	Observed reflections (*I* > 2*σ*(*I*))	1073
*c*/Å	12.0324(2)	*R*_int_	0.0556
*β*/°	99.5935(12)	*T*_min_; *T*_max_	0.4690; 0.7483
*V*/Å^3^	317.565(10)	No. of parameters	31
Single-crystal data		*R*_1_ (*F*^2^ > 2*σ*(*F*^2^))	0.0259
*a*/Å	5.4142(4)	w*R*_2_ (*F*^2^ all)	0.0484
*b*/Å	4.9471(4)	GOF	1.051
*c*/Å	12.0437(11)	Δ*ρ*_max/min_/e Å^−3^	1.62/−2.43
*β*/°	99.603(3)	CSD deposition number	2380686
*V*/Å^3^	318.07(5)		

### Single-crystal X-ray diffraction

For the single-crystal X-ray diffraction study, crystals were optically pre-selected under a polarizing microscope and positioned at the tip of Kapton micro mounts with the help of perfluorinated oil. The diffraction data were recorded at room temperature with graphite-monochromatized Mo-K_α_ radiation using a Bruker KAPPA APEX II diffractometer (Bruker AXS, Wisconsin, Madison, USA) equipped with a CCD detector. Optimization of the measurement strategy and integration were done with the diffractometer software,^33^ absorption correction with the semi-empirical method of SADABS,^34^ structure solution with SHELXT^35^ and structure refinement with SHELXL.^36^ The setting *I*2/*a* (cell choice 3) of space group No. 15 (standard setting *C*2/*c*; cell choice 1)‡‡The setting in *I*2/*a* can be transformed to the standard setting in *C*2/*c* by the transformation matrix (**P**, p): **−a − c**,**b**,**a**; 0,0,0. was chosen to emphasize the relation of the unit cell determined by Sleight *et al.* (*a* = 5.4129, *b* = 4.9471, *c* = 6.0185 Å, *β* = 99.596° with assumed space groups *C*2, *Cm* or *C*2/*m*)^22^ with the unit cell determined from the material synthesized in our study, where *c* is doubled.

Details of structure and refinement data are gathered in Table 1. Further details of the crystal structure investigation may be obtained from the joint CCDC/FIZ Karlsruhe online deposition service: https://www.ccdc.cam.ac.uk/structures/ by quoting the deposition number specified at the end of Table 1.

Bond valence sums (BVS)^37^ were calculated using the bond valence parameters provided by Krivovichev & Brown for the Pb^II^–O pair^38^ and by Mills & Christy for the Te^VI^–O pair^39^ under consideration of all oxygen atoms within a distance of 3.55 Å. Results of the BVS calculation are compiled in Table 2 together with selected bond lengths.

**Table 2:** Selected bond lengths/Å and bond valence sums (BVS)/valence units

Pb1–O1^i^	2.330(3)	Pb1–O1	3.388(3)^ix^	**BVS**	
Pb1–O1^ii^	2.330(3)	Pb1–O1	3.388(3)^x^	Pb1 (CN 10)	2.26
Pb1–O1^iii^	2.476(3)	Pb1–*E*	0.583	Te1 (CN 6)	5.89
Pb1–O1^iv^	2.476(3)	Te1–O1	1.867(3)^xiii^	O1 (CN 4; 1 Te, 3 Pb)	1.98
Pb1–O2^v^	2.956(3)	Te1–O1	1.867(3)^viii^	O2 (CN 4; 2 Te, 2 Pb)	2.09
Pb1–O2^vi^	2.956(3)	Te1–O2	1.966(2)^xiv^		
Pb1–O2^vii^	3.004(3)	Te1–O2	1.966(2)^i^		
Pb1–O2^viii^	3.004(3)	Te1–O2	1.968(3)^xiii^		
		Te1–O2	1.968(3)^viii^		

Isotypic structures were quantitatively compared using the compstru software^40^ available at the Bilbao crystallographic server.^41^

The position of the 6s^2^ electron lone pair of the Pb^II^ atom was calculated with the LPLoc software;^42^ its coordinates are given in the discussion of the crystal structure.

### Energy dispersive X-ray spectroscopy (EDX)

Several crystals of PbTeO_4_ were subjected to semiquantitative EDX measurements using a field emission scanning electron microscope (Clara Ultra High Resolution, TESCAN GmbH, Dortmund, Germany) with an energy-dispersive Ultim Max (65 mm^2^) X-ray detector-system (OXFORD Instruments NanoAnalysis, Wiesbaden, Germany) for elemental identification. The crystals were placed on a carbon plate on an aluminum holder and sputtered with carbon. Imaging and EDX measurements were performed in analysis mode at an acceleration voltage of 20 keV and a beam current of 3 nA at a working distance of 9 mm. Regarding the Pb/Te/O ratio, the values from ten measurement points were arithmetically averaged. The ESI† provides electron micrographs of the crystals with the positions of the measurement spots indicated as well as selected EDX spectra and determined compositions (Fig. S1, S2 and Table S1‡).

### Thermal analysis

Simultaneous thermal analysis (STA) measurements (thermogravimetry/differential scanning calorimetry; TG/DSC) were conducted on a Netzsch STA 449F3 instrument (Netzsch GmbH, Selb, Germany) with a ∼15 mg sample in the temperature range 25 ⇌ 725 °C (corundum crucibles, flowing argon atmosphere (3 ml min^−1^), heating/cooling rate 20 °C min^−1^). A base line correction of the TG curve was carried out by measuring the empty crucible and subtracting the data from the measurement data.

### UV/Vis spectroscopy

A diffuse reflectance spectrum of powdered PbTeO_4_ was recorded in the range of 200 to 2550 nm, using an Agilent Cary 5000 UV-Vis spectrometer equipped with an integrating sphere (DRA-2500), a D65 as standard illuminant and a 10° complementary observer. A scan rate of 600 nm min^−1^ and a data interval of 1 nm were applied and BaSO_4_ was used as white standard. The Kubelka–Munk (K–M) function was used to calculate the optical absorbance from the generated reflectance data, and the band gap was determined using Tauc-plots.^43,44^

### IR spectroscopy

Infrared spectra from powdered samples of PbTeO_4_ were collected in the spectral range of 400–1500 cm^−1^ using a Bruker Alpha Platinum FTIR-ATR spectrometer (Bruker, Billerica, USA) equipped with a 2 × 2 mm diamond ATR-crystal. A DTGS detector collected the intensity during 24 scans, and atmospheric influences were corrected *via* a reference measurement using the Opus software.^45^

### Raman spectroscopy

Raman spectra of PbTeO_4_ were recorded in the spectral range 0–1000 cm^−1^ (spectral resolution 1 cm^−1^) on a WITec 300 RAS system (WITec, Ulm, Germany) coupled with a Peltier-cooled CCD camera using the 532 nm green laser line with 0.59 mW power. For the final spectrum, 60 scans were accumulated in order to get a better signal-to-noise ratio.

### Quantum chemical calculations

The Crystal23 program package^46,47^ was used for the theoretical investigation of PbTeO_4_ utilizing the HSEsol hybrid functional for solids^48^ in combination with the Pb_pob_TZVP_rev2 basis set for lead,^49^ the Te_m-pVDZ-PP_Heyd_2005 basis set for tellurium^50^ and the O_8-411d11f_mahmoud_2013 basis set for oxygen.^51^ The Monkhorst–Pack integration scheme^52^ with a shrinking factor of the reciprocal lattice vectors of 18 was employed to ensure the correct treatment of the compact irreducible part of the Brillouin Zone (BZ). The self-consistent field (SCF) convergence criterion was set to 10^−8^ Hartree and 4.5 × 10^−4^ Hartree per Bohr for the energy and forces, respectively. The theoretically calculated crystal structure was compared with experimental data by computing X-ray powder diffraction patterns with the RIETAN-FP^53^ module as implemented in the program VESTA.^54^ A Mulliken population analysis was performed to access the partial charges of the investigated lead tellurate. The electronic band structure and the associated density of states (DOS) have been calculated at the optimized geometry in the standard *C*2/*c* setting of space group No. 15. To visualize the local electron pair distributions in the crystal structure, the electron localization function (ELF) has been calculated using the program Multiwfn^55^ and visualized using the free program package Visual Molecular Dynamics (VMD).^56^ To determine the intensities fully analytically, harmonic infrared (IR) and Raman vibrational spectra were computed through the coupled-perturbed Hartree–Fock/Kohn–Sham approach^57–59^ within Crystal23, providing a detailed analysis of associated normal modes in the crystal environment.^60^ For improved comparison, a weighted Gaussian kernel density estimation was applied to the IR bands obtained from the calculation.

## Results and discussion

### Preparation

The formation of “lead tellurate” (=PbTeO_4_) has first been described by Mathers and Graham. For the synthesis, they used a thoroughly ground mixture of Te^IV^O_2_ and Pb^IV^O_2_ (stoichiometric ratio of 1 : 1.1) heated for about two hours at 170 °C, leading to a conversion rate of Te^IV^O_2_ into the tellurate of 99.8%.^17^ However, the yield of the material supposedly produced in this way was not determined directly, but *via* telluric acid obtained by reacting the as-synthesized product with sulfuric acid in an aqueous medium and subsequent crystallization. During the present study, we have reproduced the experimental procedure (heating a 1 : 1.1 mixture of Te^IV^O_2_ and Pb^IV^O_2_ at 170 °C for two hours) and examined the resulting solid material by XRPD. All reflections could be assigned solely to the two starting materials Te^IV^O_2_ and Pb^IV^O_2_, which means that a direct reaction according to eqn (1) does not take place under these conditions.

1 PbO_2_ (s) + TeO_2_ (s) → PbTeO_4_ (s)

The apparently observed oxidation of TeO_2_ by PbO_2_ as the oxidizing agent to yield telluric acid, however, can be achieved in a highly acidic aqueous medium.

A phase of composition PbTeO_4_ obtained under ambient pressure conditions was mentioned by Young.^5^ This phase is said to have been formed when 1 : 1 mixtures of PbO and TeO_2_ were heated to 530 °C in an argon atmosphere. Apart from the reference to an earlier work^18^ and specifications of interplanar spacings, there was no further crystallographic information given on this phase.^5^

It was also claimed by Gaitán *et al.* to have obtained a phase of composition PbTeO_4_ under ambient pressure conditions.^19^ This report is somewhat confusing, as its title mentions an orthorhombic PbTeO_4_ phase, whereas the discussion refers to a tetragonal PbTeO_4_ phase. In any case, the indication of the corresponding *d* values of the observed reflections and the given unit cell parameters (*a* = 5.132, *c* = 12.862 Å) suggest that the described phase in fact is tetragonal PbTeO_3_, the composition and crystal structure of which had been determined previously (*a* = 5.304, *c* = 11.900 Å).^9^

The only reliable data on the preparation of PbTeO_4_ come from Sleight *et al.*^22^ who used a 1 : 1 mixture of PbO_2_ and TeO_2_ treated at a pressure of 3000 atm and a temperature of 700 °C. In our study, we likewise used high-pressure conditions for the successful preparation of PbTeO_4_.

Based on the partly contradictory information regarding the preparation of crystalline PbTeO_4_ given so far, it can be concluded that this phase most probably can only be obtained under high-pressure conditions.

### Composition, crystal structure and thermal behavior

The elemental composition was confirmed by EDX as PbTeO_4_ with a determined Pb/Te/O ratio of 16 ± 2 at%, 15 ± 2 at%, and 69 ± 3 at%, being close to the theoretical values of 16.67 at%, 16.67 at%, and 66.67 at% (Pb/Te/O). No other additional elements could be detected.

Whether the doubling of the unit cell relative to the previous study of PbTeO_4_ ^22^ results from the different preparation conditions (700 °C and 3000 atm for 8 hours *versus* 750 °C and 8 GPa), or from superstructure reflections unrecognized in the original study, cannot be evidenced. XRPD analysis and Rietveld refinements on PbTeO_4_ clearly revealed superstructure reflections in the low-angle area (see Fig. 1) leading to a doubling of the *c* axis. In addition, the sample composition was refined to a purity of 92%_wt_, and a minor side-phase of Pb_2_TeO_5_ ^16^ was identified. A single reflection of a yet unknown phase has been marked with an asterisk in Fig. 1.

PbTeO_4_ belongs to the family of metatellurates(vi) with general formula M^II^Te^VI^O_4_, for which only a few other representatives are known, *viz.* the first-row transition compounds CuTeO_4_,^61^ dirutile-type CoTeO_4_ ^62^ and NiTeO_4_,^63^ as well as the alkaline earth compounds CaTeO_4_,^64^ SrTeO_4_ ^64,65^ and BaTeO_4_.^66^ Although the ionic radius of 1.4 Å for Pb^II^ with CN = 10^67^ is comparable with that of 1.36 and 1.52 Å for Sr^II^ and Ba^II^, respectively, PbTeO_4_ adopts neither the SrTeO_4_ nor the BaTeO_4_ structure.

The crystal structure of PbTeO_4_ comprises four atoms in the asymmetric unit, *viz.* Pb1 on a site with site symmetry 2 (multiplicity 4, Wyckoff letter *e*), Te1 with site symmetry 1̄ (4 *d*), and two O atoms (O1, O2), each on a general position (8 *f*) of space group *I*2/*a*.

**Fig. 2:**
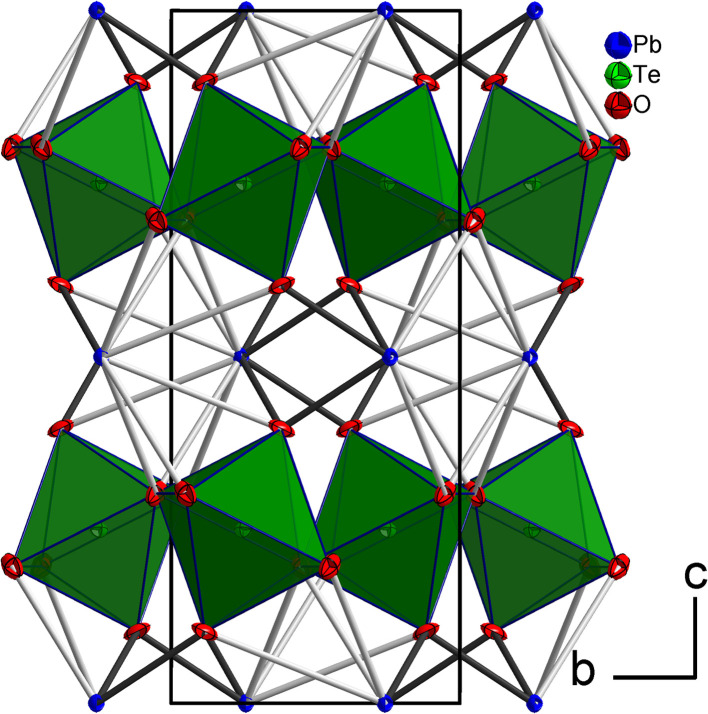
The crystal structure of PbTeO_4_ in a projection along [100]. Pb atoms are given in blue, O atoms in red, and [TeO_6_] units as green polyhedra. Short Pb–O bonds <2.70 Å are marked with black lines, and longer Pb–O bonds up to 3.50 Å as grey lines. Displacement ellipsoids are drawn at the 90% probability level.

**Fig. 3:**
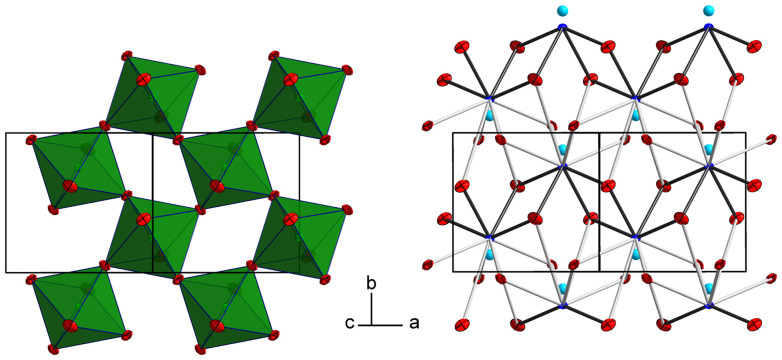
^2^_∝_[TeO_4/2_O_2/1_] layers and the intermediate Pb^II^ atoms with the coordinating oxygen atoms. Colors and displacement ellipsoids are as in Fig. 2.

The tellurium atom has an oxidation state of +VI and shows the characteristic coordination number of six within a slightly distorted octahedral coordination environment by oxygen atoms.^68^ The mean Te^VI^–O bond length of 1.934 Å is in good agreement with literature data of 1.923(30) Å.^69^ In the crystal structure, [TeO_6_] octahedra share corners to build ^2^_∝_[TeO_4/2_O_2/1_] layers extending parallel to (001) (Fig. 2 and 3). The Pb^II^ cations are located between adjacent ^2^_∝_[TeO_4/2_O_2/1_] layers with short Pb–O bonds to the terminal O atoms and with long bonds to the bridging O atoms of the ^2^_∝_[TeO_4_/_2_O_2_/_1_] layer.

For the definition of the first coordination sphere around the Pb^II^ cation in the crystal structure of PbTeO_4_, a threshold of 3.55 Å for the Pb–O bond length was used^12^ that is based on the sum of the van der Waals radii of Pb and O (2.02 and 1.52 Å, respectively).^70^ Owing to the broad Pb–O bond lengths distribution between 2.3 and 3.4 Å (Table 2), a partition into “short” Pb–O bonds less than 2.7 Å and “long” Pb–O bonds greater than this boundary up to 3.5 Å is reasonable. Consequently, the Pb^II^ cation in PbTeO_4_ shows two pairs of short and three pairs of long Pb–O bonds, resulting in a hemidirected arrangement^24^ of the ten O^2−^ ligand atoms. The mean Pb–O bond length for the [PbO_10_] polyhedron is 2.821 Å, a value that conforms with literature data (2.789(212) Å) for Pb^II^ with a coordination number of 10.^69^

**Fig. 4:**
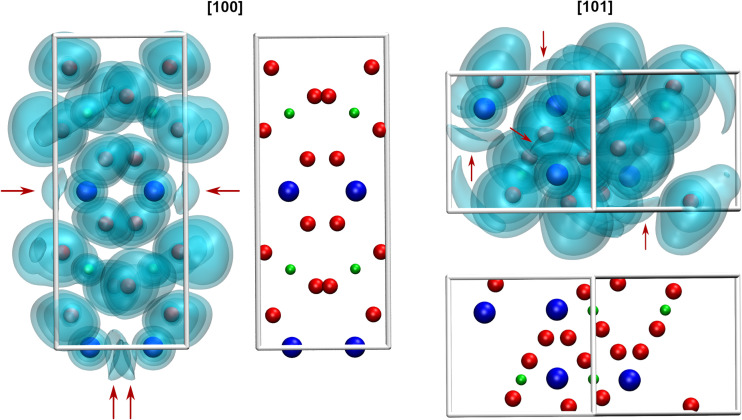
Visualization of the ELF of PbTeO_4_ along the [100] direction (left) and the [101] direction (right). Red arrows indicate the spatial extension of electron density associated with the electron lone pair *E* of the Pb^II^ atoms. Based on the calculation approach only the exact occupation of a single unit cell (four formula units of PbTeO_4_) is presented.

Due to the space requirement of the electron lone pair *E* (fractional coordinates in the unit cell: *x* = 0.75, *y* = 0.12336, *z* = 0.5 assuming a radius of 1.30 Å for *E*), it is directed towards the part of the ligand sphere with the long Pb–O bonds (Fig. 2, right), with a calculated Pb–*E* distance of 0.58 Å as obtained from the LPLoc software.^42^ The orientation of the Pb^II^ lone pair *E* was also confirmed and visualized by the computed ELF within the PbTeO_4_ unit cell. The ELF viewed along the [100] and the [101] directions is shown in Fig. 4 for isosurface values of 0.45, 0.6 and 0.75. The spatial extension of the electron lone pairs at the Pb^II^ atoms are indicated *via* red arrows. In accordance with chemical intuition and the data given above, the lone pair is oriented towards the volume of the coordination sphere where the oxygen atoms are found at longer distances.

PbTeO_4_ crystallizes isotypically with structures where the stereoactivity of the lone-pair electrons is prevalent, or which have a peculiar crystal chemistry: Bi^III^Bi^V^O_4_ (=Bi_2_O_4_),^71^ Bi^III^Sb^V^O_4_,^72^ Hg^II^Mo^VI^O_4_,^73^ Hg^II^W^VI^O_4_,^74^ β-Sb^III^Sb^V^O_4_ (=β-Sb_2_O_4_)^75^ and LiAuF_4_.^76^ It is noted that in literature there is no clear classification with respect to the designation of the corresponding structure type. Hence, Bi^III^Sb^V^O_4_, β-Sb_2_O_4_ or Hg^II^Mo^VI^O_4_ are synonymously used as such.

The similarity parameters of PbTeO_4_ and its isotypic crystal structures, as determined with the compstru program,^40^ are listed in Table 3. On the one hand, the greatest similarity in terms of *S* and *Δ* is ascribed to Bi_2_O_4_, BiSbO_4_ and β-Sb_2_O_4_. Common to all of these four crystal structures is the presence of large cations with a stereochemically active lone-pair electron *E* (Pb^II^, Sb^III^, Bi^III^) at the same site, accompanied with cations having the formal electronic configuration of a noble gas (Te^VI^, Sb^V^, Bi^V^) at the other metal site. On the other hand, the lowest similarities in terms of *S* and *Δ* pertain to HgMoO_4_, HgWO_4_, and LiAuF_4_. In case of HgMoO_4_ and HgWO_4_, the larger differences can be ascribed to the peculiar crystal chemistry of Hg^II^ with its clear preference for a linear [2 + *x*] coordination,^77^ which results in high displacements of the anions relative to Te^VI^ that prefers a more or less undistorted octahedral coordination sphere.^68^ In case of the only fluoride compound in this isotypic series, the high displacements are caused by the different ionic radii of Li^I^ and Pb^II^ and the peculiar crystal chemistry of Au^III^ with a preference for a square-planar coordination.^78^

**Table 3:** Displacements |*u*|/Å of atoms in isotypic structures and parameters of structural comparison with PbTeO_4_ as the reference structure^a^

PbTeO_4_	Bi_2_O_4_	BiSbO_4_	β-Sb_2_O_4_	HgMoO_4_	HgWO_4_	LiAuF_4_	Site symmetry
|*u*| Pb1/atom	Bi2 0.1316	Bi1 0.1044	Sb2 0.2232	Mo1 0.3493	W1 0.3498	Li1 1.1942	2
|*u*| Te1/atom	Bi1 0	Sb1 0	Sb1 0	Hg1 0	Hg1 0	Au1 0	1̄
|*u*| O1/atom	O2 0.2993	O1 0.2402	O2 0.2467	O1 0.5408	O1 0.5258	F1 0.6237	1
|*u*| O2/atom	O1 0.2358	O2 0.1272	O1 0.0799	O2 1.3575	O2 1.3205	F2 1.1144	1
*S*	0.0155	0.0108	0.0124	0.0813	0.0824	0.0813	
*d*_av._/Å	0.2003	0.1399	0.1461	0.6910	0.6737	0.7784	
*Δ*	0.108	0.078	0.054	0.641	0.598	0.546	

a Prior to the comparison, all structures were standardized with the program STRUCTURE-TIDY.^79^

**Fig. 5:**
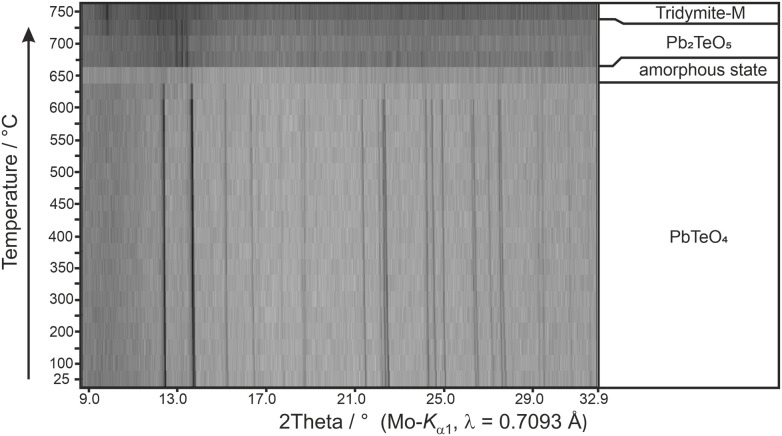
Temperature-dependent XRPD pattern of PbTeO_4_ with assigned phases and their stability ranges.

Calculated BVS values (Table 2) support the formation of Pb^II^Te^VI^O_4_ and are in expected ranges for divalent lead and hexavalent tellurium. With respect to the results of the BVS calculation, the global instability index GII^80,81^ can be used as a measure of the crystal structure strain. Hence, the GII index is an indicator of the stability and validity of a crystal structure, although its information value is not absolute but can be used as a computationally inexpensive bond valence-based metric for assigning general trends.^82^ If the GII index is smaller than 0.1 v.u., it is generally accepted that a well-balanced and stable structure is present; for strained structures a GII index between 0.1 and 0.2 v.u. is obtained, whereas a crystal structure with a GII index greater than 0.2 v.u. is considered as unstable. For the present case of PbTeO_4_, GII was computed as 0.148, revealing that the crystal structure is strained and stable. It might be speculated that the comparatively high value of GII for PbTeO_4_ is due to the fact that this phase only forms under high pressure. This assumption is also supported by the results of temperature-dependent PXRD measurements given in Fig. 5, which revealed that PbTeO_4_ shows no temperature-induced structural phase transition from a metastable high-pressure modification to a possible thermodynamically stable ambient pressure modification from room-temperature up to the end of its stability range at ∼630 °C. The non-occurrence of this transformation can also be interpreted as an indication that PbTeO_4_ cannot be produced under ambient pressure conditions and that the pressure parameter is essential for the synthesis of the lead tellurate with composition PbTeO_4_.

**Fig. 6:**
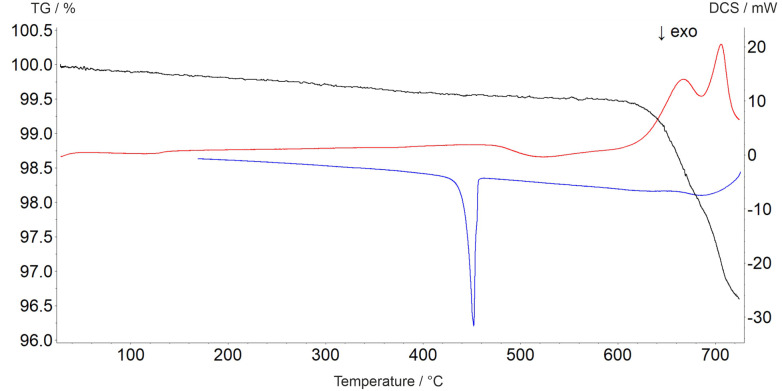
Thermal analysis curves of PbTeO_4_ (TG curve black, DSC curve on heating red, DSC curve on cooling blue).

The decomposition temperature is corroborated by complementary STA measurements (Fig. 6), which indicate the beginning of the decomposition with an onset of 625 °C. The subsequent mass loss is ≈3.5% until the end of the measurement at 725 °C and can be related to the release of oxygen according to eqn (2), which is accompanied by two endothermal effects (onset 628 °C, peak maximum 667 °C; onset 690 °C, peak maximum 706 °C). Re-cooling the sample from 725 °C to room temperature showed a sharp exothermic effect (onset 455 °C, peak maximum 452 °C), which is caused by recrystallization to the tetragonal β-modification of PbTeO_3_,^12^ as shown by subsequent XRPD measurements of the remaining sample at room temperature (Fig. S3 in the ESI†).2
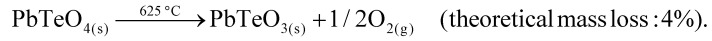


Heating the sample above 625 °C under the conditions of the high-temperature camera leads to complete amorphization at 650 °C (Fig. 5). As the temperature increases further, a significantly raised background is present and weak reflections of Pb_2_TeO_5_ ^16^ appear in the temperature range from 675 to 725 °C. This phase was already observed in the synthesis of PbTeO_4_ with a proportion of approx. 8%_wt_ (see Fig. 1) and appears to form again from the amorphous sample material at high temperatures. The corresponding reaction equation is given in eqn (3).3
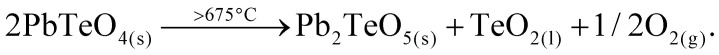


The formation of Pb_2_TeO_5_ at temperatures >675 °C as determined by temperature-dependent PXRD is not represented by the complementary STA measurement, which we attribute to the different heating rates and atmospheres used in the two measurement procedures (slow heating and holding times, atmospheric conditions for PXRD *versus* fast heating times, Ar atmosphere for STA).

Above 675 °C, TeO_2_ suggested as another decomposition product, shows no reflections, because it either forms as an amorphous phase or is already molten (melting point 733 °C).^83^ At even higher temperatures (>725 °C), only reflections of tridymite are visible, which indicates a reaction with the mark capillary and ultimately led to the breakage of the latter.

**Fig. 7:**
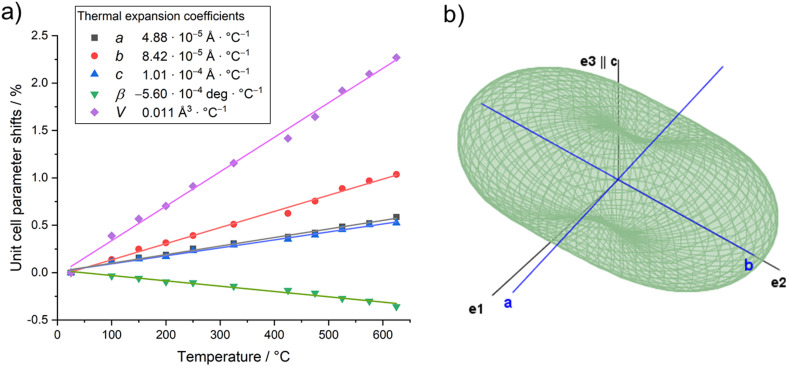
(a) Evolution of unit cell parameters with temperature; (b) three-dimensional surface of the thermal expansion tensor for PbTeO_4_ (300 °C data).

The evolution of unit cell parameters of PbTeO_4_ with temperature is depicted in Fig. 7a in form of regression lines and shows a linear increase of the unit-cell volume from room temperature up to the end of its stability region. All unit cell axes linearly increase as well with temperature, and only the monoclinic angle decreases slightly.

Based on the formalism for the infinitesimal temperature limit introduced by Paufler and Weber,^84^ the TEV software^85^ was used to calculate the components of the thermal expansion tensor αij. For the definition of the second rank tensor within the TEV program, the following orthogonalized coordinate system was used: **e3** is parallel to **c**, **e2** is parallel to b* and **e1** = **e2** × **e3**. The resulting symmetrical tensor has six independent components (α11, α22, α33, α12, α13, α23); the symmetry constraints of the monoclinic crystal system with unique axis *b* result in the components α12 and α23 being zero. After transformation to principal axes, the tensor can be simplified and is described by only three independent components (α1, α2, α3). Whereas each individual expansion coefficient shows only subtle changes with temperature, the expansion coefficient α2 has about twice the absolute value of the smallest eigenvalue α1, making the thermal expansion in PbTeO_4_ anisotropic. The dependence of eigenvalues of the thermal expansion are compiled in Fig. S4 in the ESI.† The representation of the tensor and its anisotropy in form of a surface in three-dimensional space is shown in Fig. 7b. It can be seen that the strongest expansion is along the *b* axis that exactly coincides with eigenvalue α2 and the corresponding eigenvector. The lowest expansion is perpendicular to this direction.

### UV-Vis spectroscopy

**Fig. 8:**
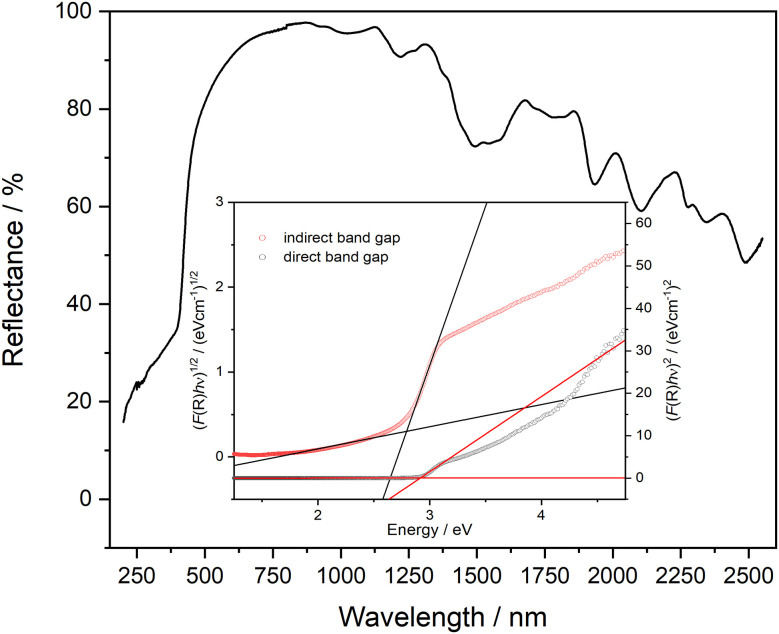
UV-Vis reflectance spectrum of PbTeO_4_. As insertion, Tauc plots to estimate the direct (2.9 eV) and indirect (2.8 eV) bandgap are given.

The UV range usually extends over the wavelength range from 380–100 nm. From around 200 nm and below, the absorption by oxygen becomes so strong that the measuring range for standard laboratory devices usually ends here. In Fig. 8 the reflectance UV-Vis spectrum of PbTeO_4_ in the range from 200–2550 nm is depicted. As can be seen, PbTeO_4_ shows a transmittance for UV light down to the lowest experimentally accessible wavelength of 200 nm. This suggests that the transmittance is also given at wavelengths shorter than 200 nm. In the range below 500 nm, there is a clear drop in reflectance and an increase in absorption. This drop also agrees very well with the calculated band gap of 2.9 eV or 427 nm. However, as the measurement clearly shows, there is no complete absorption in the UV range, so that PbTeO_4_ continues to transmit UV light. The exact mechanisms are still unclear and need to be investigated further. Highly UV-permeable materials are extremely important for technical applications such as UV/deep-UV lasers or the treatment of ultra-pure water.^86^

Additionally, the optical band gap was determined from the diffuse reflectance UV-Vis spectra *via* the Kubelka–Munk (K–M) function^44^ and Tauc plots.^43^ The K–M function (*F*(*R*)) is calculated according to *F*(*R*)_K–M_ = *K*/*S* = (1 − *R*)^2^/2*R*, in which *R* is the reflectance, *S* the scattering coefficient and *K* the absorption coefficient. To generate the Tauc plots, the factor (*F*(*R*)·*hν*)^*n*^ was plotted against photon energy. For direct and indirect band gaps, *n* was set to 0.5 and 2, respectively. By the tangent methods as shown in Fig. 8 (inset), the bandgap values *E*_g_ were determined to *E*_g(direct)_ = 2.9 eV and *E*_g(indirect)_ = 2.8 eV. Compared with the bandgap determined on basis of quantum chemical calculation (see next section) the values are almost identical.

### Quantum chemical calculations

The optimized crystal structure exhibits excellent agreement with the experimentally determined crystal structure in terms of unit cell parameters (Table S2 in the ESI†). The observed deviations are less than 1%, which can be attributed to the inherent thermal expansion of PbTeO_4_, while the energy minimization corresponds in a strict sense to a 0 K treatment. Optimized atom positions are given in Table S3 in the ESI.† The comparison of the XRPD pattern calculated from the experimentally determined structure and the optimized one also demonstrates excellent agreement as depicted in Fig. S5 in the ESI.† The partial charges (in units of the elementary charge e) of the irreducible atoms obtained from the Mulliken population analysis exhibit values of +1.432 e for Pb1, +2.876 e for Te1, −1.096 e for O1 and −1.058 e for O2.

**Fig. 9:**
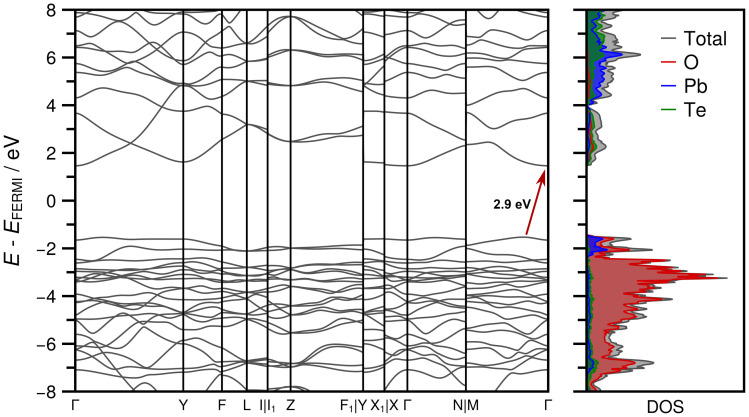
Band structure and density of states (DOS) obtained for PbTeO_4_ in the standard *C*2/*c* setting of space group No. 15 at HSEsol level of theory. The indirect band gap of 2.9 eV is marked with a red arrow.

Fig. 9 shows the band structure and the associated DOS obtained at HSEsol level. The band path through the BZ was chosen according to the convention proposed by Setyawan and Curtarolo^87^ for the standard *C*2/*c* setting. From the DOS it is evident that the bands in the valence region between −8 and −2 eV are almost exclusively formed from Pb and O orbital contributions, while only a sparse occupation is identified for Te. In the −2 eV region-bands resulting from Pb and O densities dominate and characterize the lower bound of the band gap region. The first conduction bands are more or less equally derived from unoccupied Pb, Te, and O states. The indirect band gap of 2.9 eV indicates that PbTeO_4_ is a wide band gap semiconductor, analogous to the first row metatellurate(vi) CoTeO_4_.^62^

**Fig. 10:**
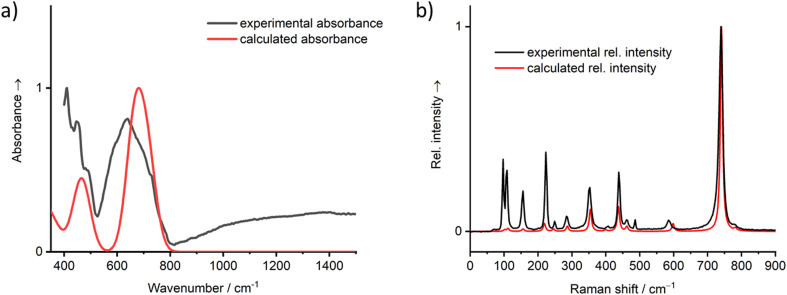
Vibrational spectra (a) IR, (b) Raman of PbTeO_4_ calculated on basis of the coupled-perturbed Hartree–Fock/Kohn–Sham approach in comparison with the measured spectra.

The calculated IR and Raman vibrational spectra on basis of the energy minimized crystal structure are depicted in Fig. 10 and show good agreement with the recorded spectra. A full set of calculated vibrational modes is compiled in the ESI, Table S3.† In the IR spectrum, two areas of overlapping strong bands are clearly distinguishable. The first region is located between 400 and 500 cm^−1^, where the ν_5_ vibrations of the [TeO_6_] octahedra and lattice vibrations are typically located. The second region between 500 and 800 cm^−1^ typically covers the ν_2–4_ vibrations, which are not clearly resolved. The total symmetric ν_1_ mode is of low intensity and not observed in the IR spectrum but very prominent in the Raman spectrum at ∼740 cm^−1^. In general, the Raman spectrum of PbTeO_4_ between 400 and 800 cm^−1^ is similar to that of CoTeO_4_,^88^ which is not surprising since the octahedral [TeO_6_] units are arranged in the same type of ^2^_∝_[TeO_4/2_O_2/1_] layers in both crystal structures. The ν_1_ mode in CoTeO_4_ likewise shows the strongest intensity but is redshifted to 705 cm^−1^. Values of 710 and 763 cm^−1^ were reported for the corresponding bands in the Raman spectrum of the rare mineral xocomecatlite.^89^ Although no crystal structure determination has yet been carried out for this mineral so far, the chemical composition of Cu_3_(Te^VI^O_4_)(OH)_4_ suggests the existence of similarly structured metatellurate TeO_4_^2−^ anions.

## Conclusion

Lead telluride and the importance of knowing about possible surface oxidation layers are of great importance, as described in the introduction. In this context, we were able to determine and analyze the crystal structure of PbTeO_4_ from single-crystal X-ray diffractometer data for the first time. From these data it was possible to clearly assign the oxidation states as Pb^II^ and Te^VI^. Previously, the only reliable data on the preparation of PbTeO_4_ came from the study of Sleight *et al.*,^22^ who produced the material at a pressure of 3000 atm. In our study, we used multianvil high-pressure conditions for the successful preparation of well-crystallized PbTeO_4_. Additional evaluation of the global instability index GII and temperature-dependent powder X-ray analysis confirmed the assumption that the parameter pressure is required for the formation of crystalline PbTeO_4_. The compound is a UV transparent material down to a wavelength of 200 nm and below, as indicated by UV-Vis measurements. From Tauc plots, the experimental band gap was determined to be *E*_g(direct)_ = 2.9 eV and *E*_g(indirect)_ = 2.8 eV. The property measurements were supplemented by quantum chemical calculations at DFT level. As suggested by previous studies^90–93^ and supported by the optimized unit cell parameters as well as the calculated powder diffraction patterns, the range-separated hybrid functional HSEsol exhibits exceptional performance in the treatment of solid-state oxide systems and enables the characterization of the Mulliken partial charges, the electronic band structure, the density of states as well as the vibrational properties of PbTeO_4_. The centrosymmetric crystal structure of PbTeO_4_ precludes an application regarding SHG, piezo-, pyro- or ferroelectric properties.

## Data availability

Crystallographic data for PbTeO_4_ has been deposited at the joint CCDC/FIZ Karlsruhe online deposition service: https://www.ccdc.cam.ac.uk/structures/ and can be obtained by quoting the deposition number 2380686.‡

## Author contributions

Matthias Weil: conceptualization, formal analysis, investigation, visualization, and writing – original draft. Gunter Heymann: conceptualization, formal analysis, investigation, funding acquisition, supervision, visualization, and writing. Thomas S. Hofer: formal analysis, investigation, supervision, visualization, and writing. Michael Hladik, Felix R. S. Purtscher, Armin Penz: formal analysis, investigation, visualization, and writing.

## Conflicts of interest

There are no conflicts to declare.

## Supplementary Material

DT-053-D4DT02697G-s001

DT-053-D4DT02697G-s002
