# Supplementary material for: High-pressure synthesis and crystal structure analysis of PbTeO4, a UV transparent material
Source: Dalton Trans. 2024 Oct 31;53(48):19214–25. doi: 10.1039/d4dt02697g (PMC11563208; doi:10.1039/d4dt02697g)
Supplement: DT-053-D4DT02697G-s001 [file DT-053-D4DT02697G-s001.pdf]

Supplementary Information (SI) for Dalton Transactions.
This journal is © The Royal Society of Chemistry 2024

Electronic Supporting Information

High-pressure synthesis and crystal structure analysis of $PbTeO_4$, a UV transparent material

Michael Hladik, [1] Armin Penz, [1] Felix R. S. Purtscher, [1] Thomas S. Hofer, [1] Gunter Heymann, [1]* Matthias Weil [2]*

1) Department of General, Inorganic and Theoretical Chemistry
Universität Innsbruck
Center for Chemistry and Biomedicine
Innrain 80-82
6020 Innsbruck / Austria

2) Institute for Chemical Technologies and Analytics
Division of Structural Chemistry
TU Wien
Getreidemarkt 9/E164-05-1
1060 Vienna / Austria

Table of Contents

Figures

1. Electron micrographs of $PbTeO_4$.
2. Electron micrograph of $PbTeO_4$ with selected spots for measurement and corresponding EDX spectra.
3. XRPD measurement (black) of the product obtained after thermal analysis measurement. The red curve corresponds to database entry of tetragonal $PbTeO_3$.
4. Dependence of eigenvalues of the thermal expansion for $PbTeO_4$.
5. Calculated XRPD pattern after energy minimization (red) compared to the data simulated from the experimentally determined single crystal structure (black).

Tables

1. EDX measurements of $PbTeO_4$. Determined compositions based on selected measurement spots.
2. Comparison of the optimized unit cell parameters obtained for $PbTeO_4$ at HSEsol level of theory with the experimentally determined values at 295 K.
3. Optimized atom positions (given in fractional coordinates) of $PbTeO_4$ in the *I*2/*a* setting at HSEsol level of theory.
4. Complete list of calculated vibrational modes of $PbTeO_4$ at HSEsol level of theory. The associated energy is provided in units of frequency *v* and the irreducible representation is abbreviated as Irrep. All possible atom pairs are calculated based on their van der Waals radii. The relative motion for each pair of bonded atoms was decomposed into three components: i) stretching vibration (S) along the A-B bond, ii) in-plane angle bending (B) involving a third atom C and iii) out-of-plane angle bending (O) involving a third atom C. The classification of the individual motions is based on a relative contribution greater than 85 % for bond stretching and in-plane angle bending. Collective vibrational motions of entire structural elements are indicated with an R. In any other case, the motion is classified as out-of-plane angle bending. The associated unit cell and respective atom positions in fractional coordinates are given in the *C*2/*c* setting at the end of the Table.

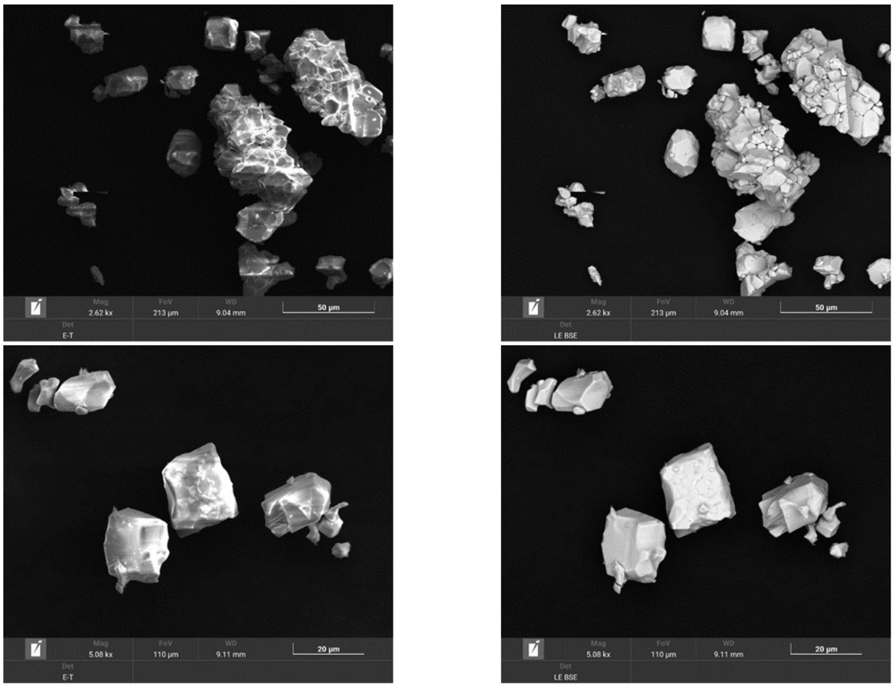

Fig. S1. Electron micrographs of $PbTeO_4$.

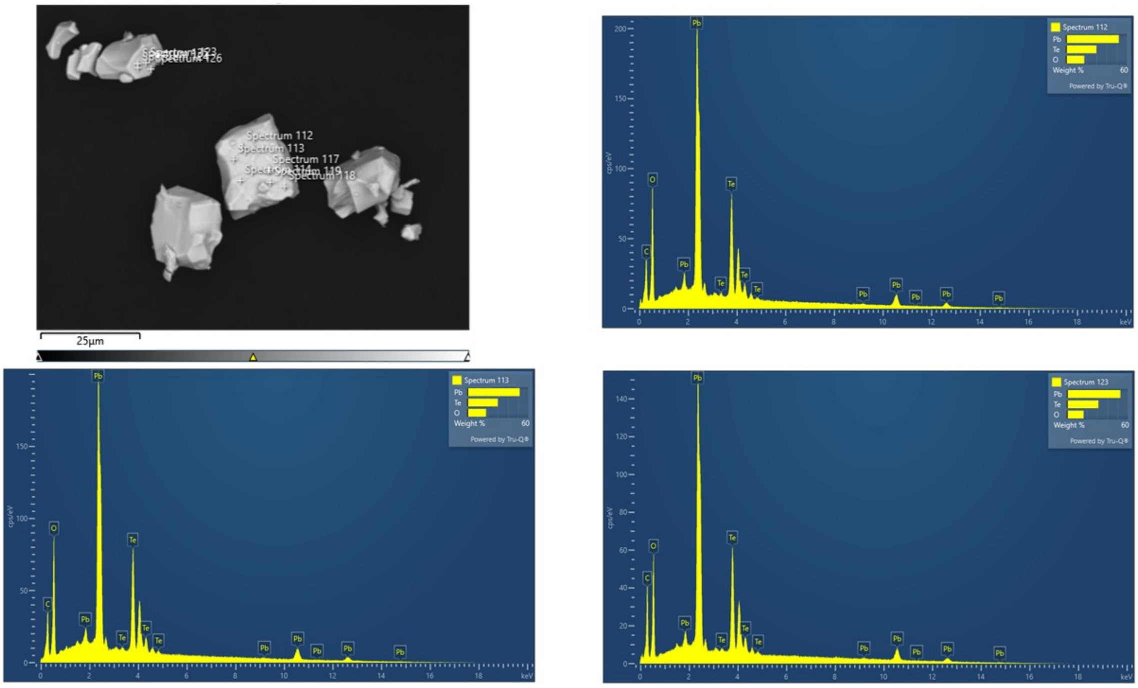

Fig. S2. Electron micrograph of $PbTeO_4$ with selected spots for measurement and corresponding EDX spectra.

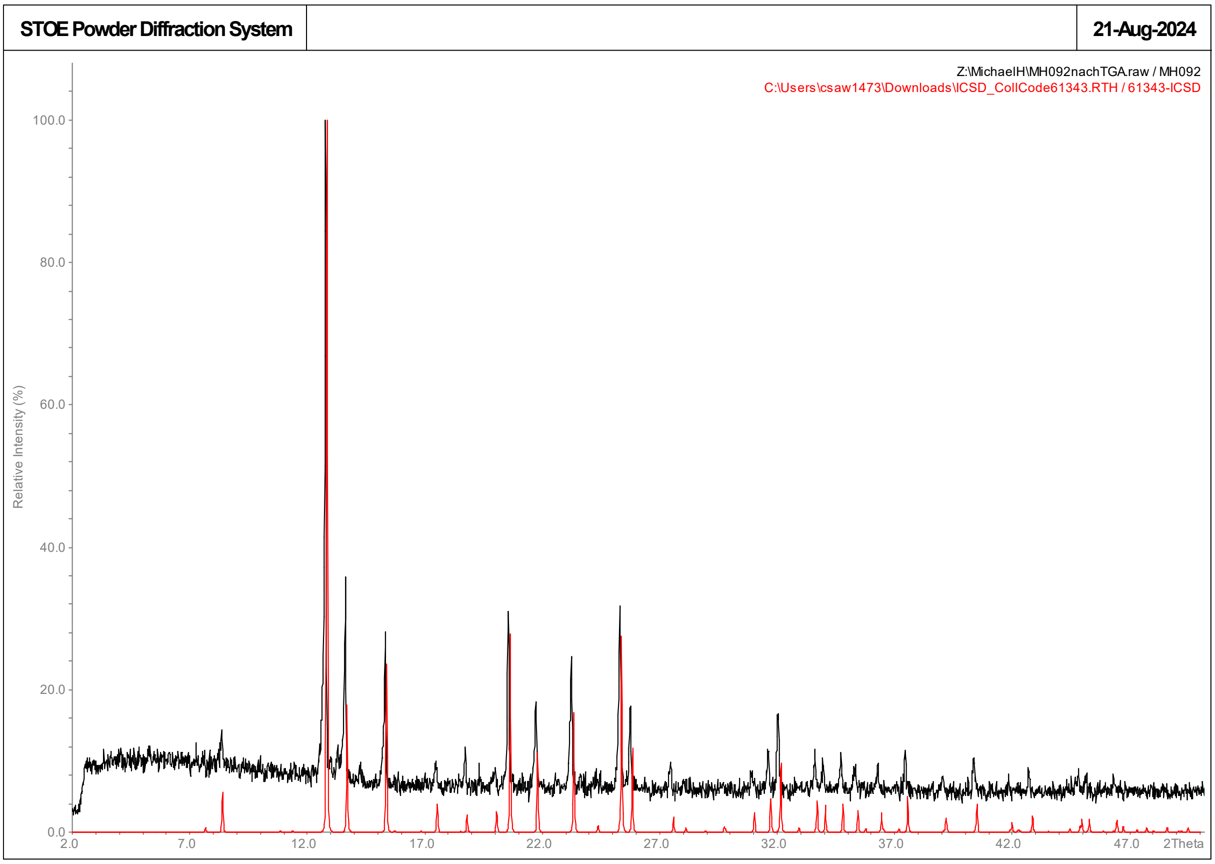

Fig. S3. XRPD measurement (black) of the product obtained after thermal analysis measurement. The red curve corresponds to database entry of tetragonal $PbTeO_3$.

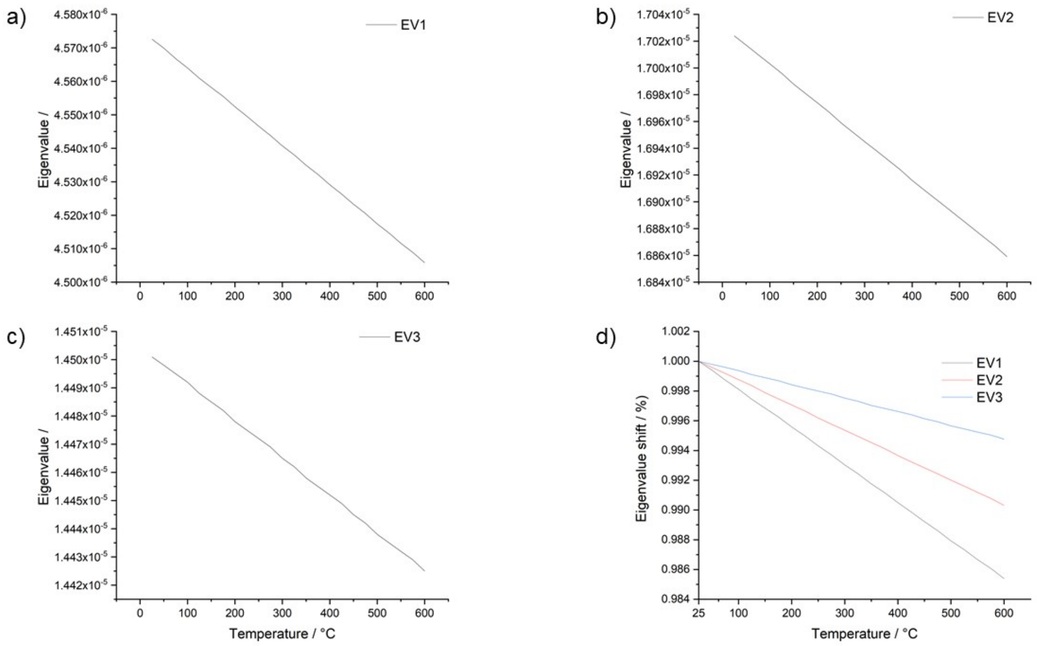

Fig. S4. Dependence of eigenvalues of the thermal expansion for $PbTeO_4$.

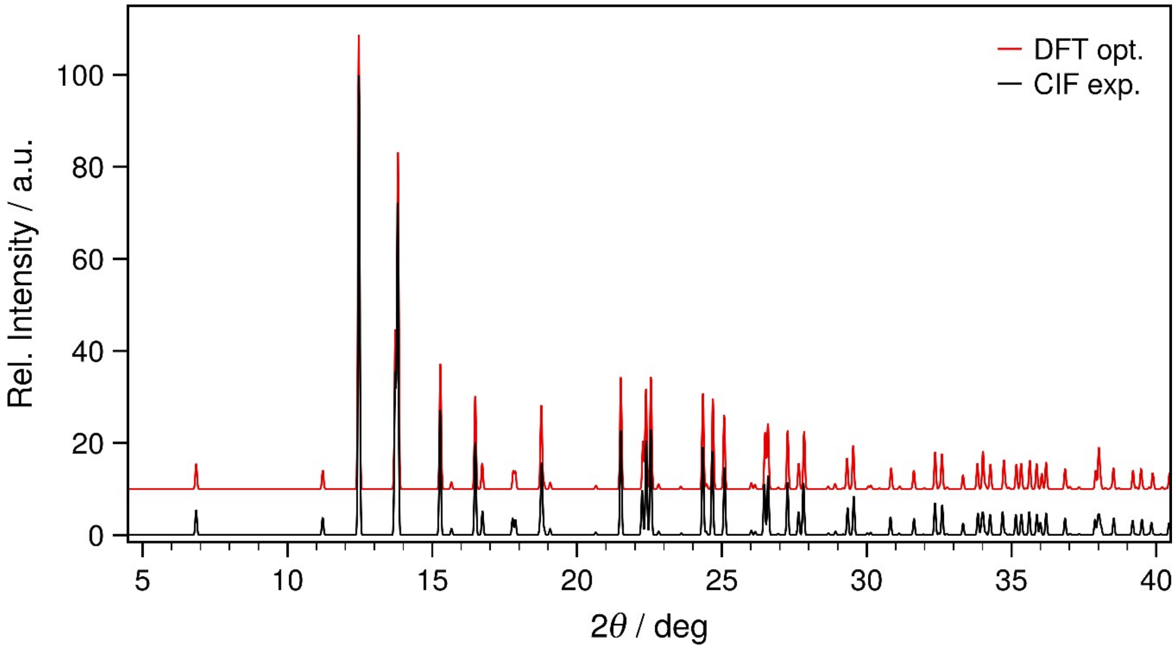

Fig. S5. Calculated XRPD pattern after energy minimization (red) compared to the data simulated from the experimentally determined single crystal structure (black).

Table S1. EDX measurements of $PbTeO_4$. Determined compositions based on selected measurement spots.

**Spectrum 112**	Element	Signal Type	Line	Wt%	Wt% Sigma	Atomic %	Standard
	O	EDS	K series	18.01	0.23	69.86	SiO_2
	Te	EDS	L series	29.89	0.24	14.54	HgTe
	Pb	EDS	M series	52.10	0.29	15.61	PbTe
	Total			100.00		100.00	
**Spectrum 113**	Element	Signal Type	Line	Wt%	Wt% Sigma	Atomic %	Standard
	O	EDS	K series	18.45	0.23	70.44	SiO_2
	Te	EDS	L series	29.97	0.24	14.35	HgTe
	Pb	EDS	M series	51.58	0.29	15.21	PbTe
	Total			100.00		100.00	
**Spectrum 123**	Element	Signal Type	Line	Wt%	Wt% Sigma	Atomic %	Standard
	O	EDS	K series	16.31	0.22	67.21	SiO_2
	Te	EDS	L series	31.00	0.25	16.02	HgTe

Pb	EDS	M series	52.70	0.29	16.77	PbTe
Total			100.00		100.00	

Table S2. Comparison of the optimized unit cell parameters obtained for $PbTeO_4$ at HSEsol level of theory with the experimentally determined values at 295 K.

Method	*a* / Å	*b* / Å	*c* / Å	*β* / °	*V* / Å^3
Single-crystal	5.4142(4)	4.9471(4)	12.0437(11)	99.603(3)	318.07(5)
Powder	5.41173(10)	4.94608(9)	12.0324(2)	99.5935(12)	317.565(10)
HSEsol	5.4123	4.9476	12.045	99.721	317.91

Table S3. Optimized atom positions (given in fractional coordinates) of $PbTeO_4$ in the *I*2/*a* setting at HSEsol level of theory.

Atom	*x*	*y*	*z*
Pb1	0.25	-0.25849	0
Te1	0.25	0.25	-0.25
O1	0.33851	0.11365	-0.10387
O2	0.02289	-0.44769	0.19587

Table S4. Complete list of calculated vibrational modes of $PbTeO_4$ at HSEsol level of theory. The associated energy is provided in units of frequency *v* and the irreducible representation is abbreviated as Irrep. All possible atom pairs are calculated based on their van der Waals radii. The relative motion for each pair of bonded atoms was decomposed into three components: i) stretching vibration (S) along the A-B bond, ii) in-plane angle bending (B) involving a third atom C and iii) out-of-plane angle bending (O) involving a third atom C. The classification of the individual motions is based on a relative contribution greater than 85 % for bond stretching and in-plane angle bending. Collective vibrational motions of entire structural elements are indicated with an R. In any other case, the motion is classified as out-of-plane angle bending. The associated unit cell and respective atom positions in fractional coordinates are given in the *C*2/*c* setting at the end of the Table.

**Mode**	***v* / cm^{-1}**	**Irrep**	**Type**	**Atom A**	**Atom B**	**Atom C**
1	55.2075	B_g	R	Pb2	Pb1	O5
			R	Pb2	Pb1	O8
			R	Pb2	Pb1	O6
			R	Pb2	Pb1	O7
2	61.9618	B_u	R	O5	Pb1	Pb2
			R	O6	Pb1	Pb2
			R	O7	Pb1	O6
			R	O8	Pb1	O5
			R	O5	Pb2	O8
			R	O6	Pb2	O7
			R	O7	Pb2	Pb1
			R	O8	Pb2	Pb1
3	99.9834	A_u	R	O5	Pb1	O6
			R	O6	Pb1	O5
			R	O7	Pb1	O8
			R	O8	Pb1	O7

			R	O5	Pb2	O6
			R	O6	Pb2	O5
			R	O7	Pb2	O8
			R	O8	Pb2	O7
4	101.689	B_g	S	Pb2	Pb1	Pb2
			S	Pb2	Pb1	Pb2
			S	Pb2	Pb1	Pb2
			S	Pb2	Pb1	Pb2
5	111.5628	A_g	B	Pb2	Pb1	Pb2
			B	Pb2	Pb1	Pb2
			B	Pb2	Pb1	Pb2
			B	Pb2	Pb1	Pb2
6	121.1118	B_u	B	Pb1	O5	Te3
			B	Pb1	O6	Te4
			O	Pb1	O7	Te3
			O	Pb1	O8	Te4
			O	Pb2	O5	Te3
			O	Pb2	O6	Te4
			B	Pb2	O7	Te3
			B	Pb2	O8	Te4
7	155.4987	A_g	B	O9	Te3	O12
			O	O10	Te3	O9
			B	O11	Te3	O12
			O	O12	Te3	O9
			O	O9	Te4	O10
			B	O10	Te4	O9
			O	O11	Te4	O10
			B	O12	Te4	O9
8	195.4488	A_u	B	Te3	O10	Te4
			B	Te3	O12	Te4
			B	Te4	O9	Te3
			B	Te4	O11	Te3
9	206.1675	B_u	B	O5	Pb1	Pb2
			B	O6	Pb1	Pb2
			B	O7	Pb1	O6
			B	O8	Pb1	O5
			B	O5	Pb2	O8
			B	O6	Pb2	O7
			B	O7	Pb2	Pb1
			B	O8	Pb2	Pb1
			B	O5	Te3	O9
			B	O7	Te3	O9
			B	O6	Te4	O12
			B	O8	Te4	O10
10	218.7505	A_g	B	O5	Pb1	Pb2
			B	O6	Pb1	Pb2
			B	O7	Pb1	O6
			B	O8	Pb1	O5
			B	O5	Pb2	O8

			B	O6	Pb2	O7
			B	O7	Pb2	Pb1
			B	O8	Pb2	Pb1
			B	O5	Te3	O9
			B	O7	Te3	O9
			B	O6	Te4	O12
			B	O8	Te4	O12
11	245.6428	B_g	B	O5	Pb1	O6
			B	O6	Pb1	O5
			B	O7	Pb1	O6
			B	O8	Pb1	O5
			B	O5	Pb2	O8
			B	O6	Pb2	O7
			B	O7	Pb2	O8
			B	O8	Pb2	O7
			B	O5	Te3	O9
			B	O7	Te3	O9
			B	O6	Te4	O12
			B	O8	Te4	O12
12	247.8098	A_u	O	O5	Te3	O9
			O	O7	Te3	O9
			B	O9	Te3	O12
			B	O11	Te3	O12
			O	O6	Te4	O12
			O	O8	Te4	O12
			B	O10	Te4	O9
			B	O12	Te4	O9
13	254.6722	B_u	B	O9	Te3	O12
			B	O11	Te3	O12
			B	O10	Te4	O9
			B	O12	Te4	O9
14	273.4607	A_u	O	O10	Te3	O9
			O	O12	Te3	O9
			O	O9	Te4	O10
			O	O11	Te4	O10
15	286.2286	B_g	O	O9	Te3	O12
			B	O10	Te3	O5
			O	O11	Te3	O12
			B	O12	Te3	O5
			B	O9	Te4	O6
			O	O10	Te4	O9
			B	O11	Te4	O6
			O	O12	Te4	O9
16	293.9963	A_u	O	O5	Te3	O9
			O	O7	Te3	O9
			B	O9	Te3	O12
			B	O10	Te3	O5
			B	O11	Te3	O12
			B	O12	Te3	O5

			O	O6	Te4	O12
			O	O8	Te4	O12
			B	O9	Te4	O6
			B	O10	Te4	O9
			B	O11	Te4	O6
			B	O12	Te4	O9
17	294.404	B_u	O	O10	Te3	O9
			O	O12	Te3	O9
			O	O9	Te4	O10
			O	O11	Te4	O10
18	354.3651	A_g	B	O5	Pb1	Pb2
			B	O6	Pb1	Pb2
			S	O7	Pb1	
			S	O8	Pb1	
			S	O5	Pb2	
			S	O6	Pb2	
			B	O7	Pb2	Pb1
			B	O8	Pb2	Pb1
			B	Te3	O5	Pb2
			B	Te3	O7	Pb1
			B	Te4	O6	Pb2
			B	Te4	O8	Pb1
19	355.6528	B_u	B	Te3	O5	Pb1
			B	Te3	O7	Pb2
			B	Te4	O6	Pb1
			B	Te4	O8	Pb2
20	399.3387	B_g	B	O9	Te3	O12
			O	O10	Te3	O9
			B	O11	Te3	O12
			O	O12	Te3	O9
			O	O9	Te4	O10
			B	O10	Te4	O9
			O	O11	Te4	O10
			B	O12	Te4	O9
21	423.2132	A_u	B	Te3	O9	Te4
			B	Te3	O11	Te4
			B	Te4	O10	Te3
			B	Te4	O12	Te3
22	436.9384	A_g	B	O9	Te3	O5
			B	O10	Te3	O5
			B	O11	Te3	O5
			B	O12	Te3	O5
			B	O9	Te4	O6
			B	O10	Te4	O6
			B	O11	Te4	O6
			B	O12	Te4	O8
23	450.7137	A_u	B	Te3	O5	Pb2
			B	Te3	O7	Pb1
			B	Te4	O6	Pb2

			B	Te4	O8	Pb1
24	461.7121	B_g	B	Te3	O9	Te4
			B	Te3	O10	Te4
			B	Te3	O11	Te4
			B	Te3	O12	Te4
			B	Te4	O9	Te3
			B	Te4	O10	Te3
			B	Te4	O11	Te3
			B	Te4	O12	Te3
25	468.5529	B_u	B	O9	Te3	O5
			B	Te3	O10	Te4
			B	O11	Te3	O5
			B	Te3	O12	Te4
			B	Te4	O9	Te3
			B	O10	Te4	O6
			B	Te4	O11	Te3
			B	O12	Te4	O8
26	598.1403	B_g	S	Pb1	O5	Te3
			S	Pb1	O6	Te4
			B	Pb1	O7	Te3
			B	Pb1	O8	Te4
			B	Pb2	O5	Te3
			B	Pb2	O6	Te4
			S	Pb2	O7	Te3
			S	Pb2	O8	Te4
			S	O5	Te3	
			S	O7	Te3	
			S	O6	Te4	
			S	O8	Te4	
27	613.4356	A_g	S	O9	Te3	
			S	Te3	O10	Te4
			S	O11	Te3	
			S	Te3	O12	Te4
			S	Te4	O9	Te3
			S	O10	Te4	
			S	Te4	O11	Te3
			S	O12	Te4	
28	658.6918	B_u	S	Te3	O9	Te4
			S	O10	Te3	
			S	Te3	O11	Te4
			S	O12	Te3	
			S	O9	Te4	
			S	Te4	O10	Te3
			S	O11	Te4	
			S	Te4	O12	Te3
29	674.588	A_u	S	O9	Te3	
			S	Te3	O10	Te4
			S	O11	Te3	
			S	Te3	O12	Te4

			S	Te4	O9	Te3
			S	O10	Te4	
			S	Te4	O11	Te3
			S	O12	Te4	
30	716.7821	B_u	S	O5	Te3	
			S	O7	Te3	
			S	O6	Te4	
			S	O8	Te4	
31	738.2594	A_u	S	O5	Te3	
			S	O7	Te3	
			S	O6	Te4	
			S	O8	Te4	
32	740.8657	A_g	S	Pb1	O5	Te3
			S	Pb1	O6	Te4
			B	Pb1	O7	Te3
			B	Pb1	O8	Te4
			B	Pb2	O5	Te3
			B	Pb2	O6	Te4
			S	Pb2	O7	Te3
			S	Pb2	O8	Te4
			S	O5	Te3	
			S	O7	Te3	
			S	O6	Te4	
			S	O8	Te4	
33	782.3425	B_g	S	O9	Te3	
			S	Te3	O10	Te4
			S	O11	Te3	
			S	Te3	O12	Te4
			S	Te4	O9	Te3
			S	O10	Te4	
			S	Te4	O11	Te3
			S	O12	Te4	

Unit Cell:	a / Å	b / Å	c / Å	β / °	V / Å^3
	12.3435	4.9476	5.4123	105.89	317.911

Atom (Index)	x	y	z
Pb1(1)	0.25849	0.25849	0.25000
Pb(1)	-0.25849	-0.25849	-0.25000
Te1(1)	-0.50000	0.00000	0.00000
Te2(1)	0.00000	-0.50000	0.50000
O1(1)	-0.21752	-0.00978	0.05762
O2(1)	-0.00978	-0.21752	0.44238
O3(1)	0.21752	0.00978	-0.05762
O4(1)	0.00978	0.21752	-0.44238
O5(2)	0.35645	-0.25183	0.32702

O6(2)	-0.25183	0.35645	0.17298
O7(2)	-0.35645	0.25183	-0.32702
O8(2)	0.25183	-0.35645	-0.17298
